# Age-Related Macular Degeneration: An Exponentially Emerging Imminent Threat of Visual Impairment and Irreversible Blindness

**DOI:** 10.7759/cureus.39624

**Published:** 2023-05-29

**Authors:** Madhurima Chaudhuri, Yusra Hassan, Pansy Prardhana Sinha Bakka Vemana, Muktha S Bellary Pattanashetty, Zain U Abdin, Humza F Siddiqui

**Affiliations:** 1 Department of Ophthalmology, Medical College and Hospital, Kolkata, IND; 2 Ophthalmology, University of Illinois at Chicago, Chicago, USA; 3 Department of Ophthalmology, Mayo Hospital Lahore, Lahore, PAK; 4 Department of Ophthalmology, Kurnool Medical College, Kurnool, IND; 5 Department of Ophthalmology, Akaki Tsereteli State University, Kutaisi, GEO; 6 Department of Medicine, District Head Quarter Hospital, Faisalabad, PAK; 7 Department of Medicine, Jinnah Sindh Medical University, Karachi, PAK

**Keywords:** anti-vegf, drusen, oct (optical coherence tomography), age related macular degeneration, neovascular age-related macular degeneration

## Abstract

Age-related macular degeneration (AMD) is a significant cause of blindness globally. With the exponential rise in the aging population, AMD is the third leading cause of visual impairment worldwide. Neovascular AMD (nAMD; Wet AMD) and geographical atrophy (GA, late-stage dry AMD) are the advanced AMD accountable for substantial cases of visual deterioration among the elderly. Our review of the literature depicted that notable risk factors include cigarette smoking, nutritional elements, cardiovascular disorders, and genetic markers, including genes regulating complement, lipid, and angiogenic pathways. Some studies have suggested a relative decline in the proportion of AMD cases in the last two decades attributable to novel diagnostic and therapeutic modalities. Accurate diagnosis is the result of a combination of clinical examination and imaging techniques, including retinal photography, angiography, and optical coherence tomography. The incorporation of dietary antioxidant supplements, explicitly lutein, slows the progression of the disease in advanced stages. The induction of vascular endothelial growth factor (VEGF) inhibitors in the treatment of neovascular AMD, often combined with other modalities, has shown an immensely favorable prognosis. Research to integrate gene therapy and regenerative techniques using stem cells is underway to further mitigate AMD-associated morbidity. It is imperative to establish screening and therapeutic guidelines for AMD to curtail the future social and financial burden and improve the diminishing quality of life among the elderly.

## Introduction and background

Age-related macular degeneration (AMD) is a chronic progressive eye disorder and a salient cause of irreversible blindness among the elderly population worldwide. The disease has become more overtly apparent in the last decade because of the exponential rise in life expectancy across the globe. Macula is a specialized circular region in the center of the posterior retina, approximately 5.5 mm in diameter, and located 0.53-0.8 mm inferior to the optic disc’s center. The middle of the macula incorporates a tiny, central pit called the fovea centralis. The macula is highly saturated in cone photoreceptors and is the area of highest visual distinction. Fovea is responsible for the high acuity center vision. Macula is most susceptible to damage with direct light irradiation and, hence, is the major contributor to severe progressive vision loss in adults over 55 years of age [1,2,3].

**Figure 1:**
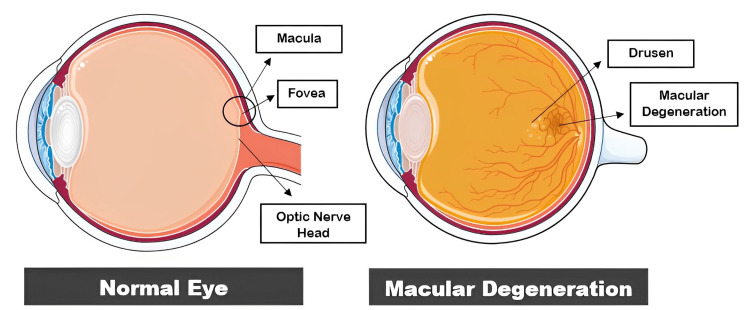
Normal eye anatomy versus macular degeneration. Parts of the figure were drawn using pictures from Servier Medical Art. Servier Medical Art by Servier is licensed under a Creative Commons Attribution 3.0 Unported License (https://creativecommons.org/licenses/by/3.0/).

**Figure 2:**
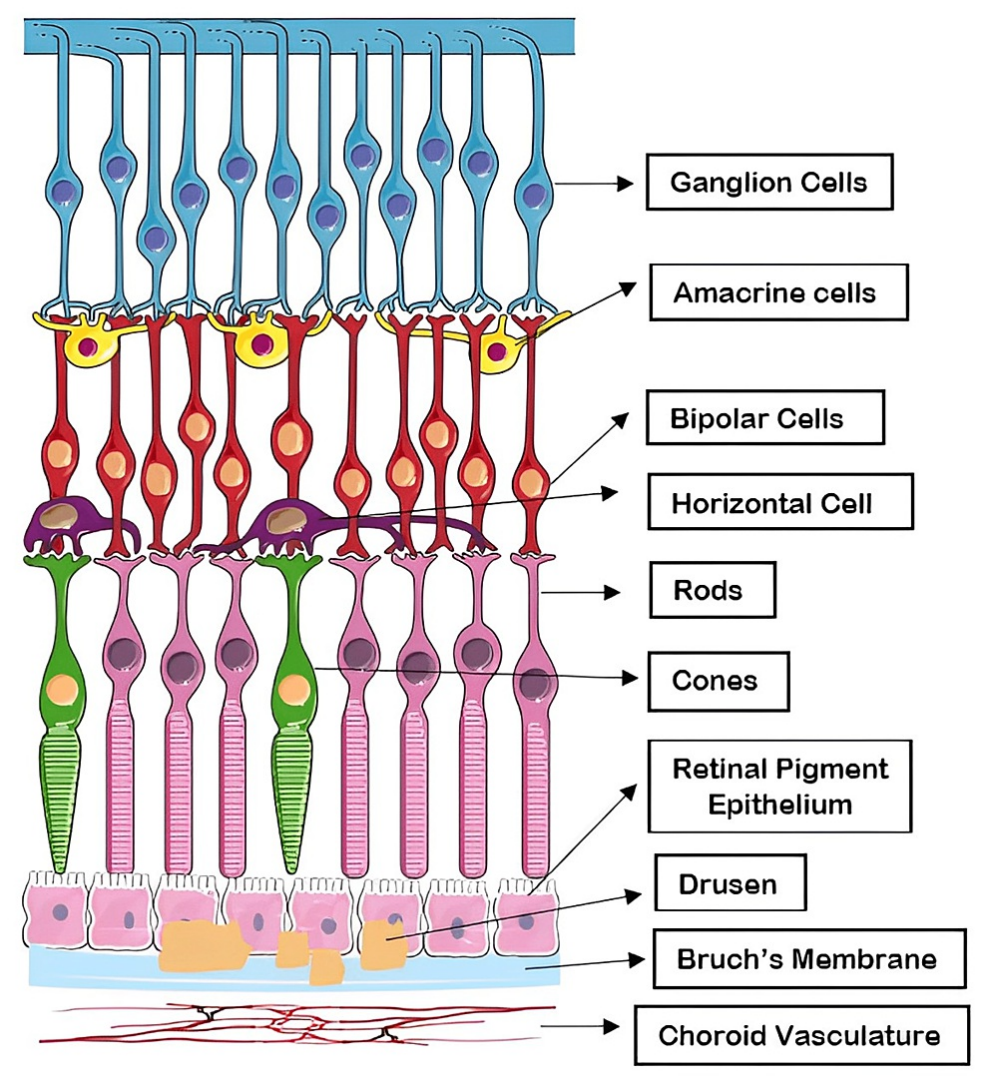
Retinal cell layers: drusen between RPE and BrM. Parts of the figure were drawn using pictures from Servier Medical Art. Servier Medical Art by Servier is licensed under a Creative Commons Attribution 3.0 Unported License (https://creativecommons.org/licenses/by/3.0/). RPE, retinal pigment epithelium; BrM, Bruch's membrane

AMD causes pathologic alterations to the macula's deeper retinal layers and the surrounding vasculature, which impairs central vision (Figures 1-2). AMD is clinically classified into two groups: dry AMD and wet AMD. Dry AMD is categorized as a lack of serum or blood leakage. The development of retinal deposits, known as drusen, is a characteristic sign of AMD and can be the first indication that the disease is in its *dry* stage. Eye symmetry imbalance, blurred vision, disorganized macular pigmentation in both eyes, the loss of the foveal reflex, and posterior poles with variable yellowish-white drusen between Bruch's membrane (BrM) and retinal pigment epithelium (RPE) are its distinguishing features. Geographical atrophy (GA) of the posterior retina is the advanced stage of dry AMD cases. A disciform scar is the ultimate stage of advanced AMD [4]. The most frequent form of AMD, known as dry AMD, can transform into *wet* or neovascular AMD (nAMD), in which central choroidal neovascular membranes (CNVs) can cause bleeding and exudation in the retina leading to significant vision loss [5]. Age, cigarette smoking, an elevated body mass index, family history, and comorbid conditions, including hypertension and hypercholesterolemia, have all been frequently identified as risk factors for AMD in epidemiological research over the past few years [6-8]. Dry AMD accounts for about 80% to 85% of cases and generally has a favorable visual prognosis, whereas wet AMD constitutes the remaining 15% to 20% of the cases and contributes to 80% of severe vision loss attributed to AMD [5,9].

In 2020, about 200 million people were affected by AMD globally. By the year 2040, around 288 million people are estimated to be affected by the disease. The disorder accounts for nearly 9% of all cases of blindness worldwide [10,11]. Two meta-analyses conducted on Asian and European populations aged 40 to 79 years revealed that late AMD had a similar prevalence of 0.56% and 0.59%, respectively. However, earlier signs of AMD were less frequent among Asians (6.8%) in comparison to Europeans (8.8%) [12-14]. A study depicted the prevalence rate of AMD as 12.33% among Europeans, 7.38% among Asians, and 7.53% among Africans. Asia, which comprises about half of the world’s population, is predicted to reach 113 million cases of AMD by the year 2040 [1,4]. A study performed in Germany showed an increase of 23% and 36% in the number of cases of early and late AMD, respectively, in 15 years between 2002 and 2017 [10]. The prevalence of early-stage AMD was 18.34 million (11.64%) and late-stage AMD was 1.49 million (0.94%) in the US population aged 40 and above in 2019 [15]. AMD is estimated to share 54.4% of visual impairment and 22.9% of blindness burden among Caucasian ethnicity in the United States [16]. Late AMD causes blindness in nearly 11 million people globally [4]. According to the World Health Organization (WHO), the majority of the patients affected by vision impairment and blindness are aged above 50 years and about 8 million of the cases are attributable to AMD, predominantly in high-income countries [17,18]. In 2015, AMD was the fourth most common cause of blindness globally (5.8% of blind individuals) and the third leading cause of moderate-to-severe vision impairment (MSVI) (3.9% of visually impaired individuals) [19]. The advent of therapeutic modalities, including intravitreally injected vascular endothelial growth factor (VEGF) inhibitors has resulted in a marked reduction in the prevalence rate of blindness in the past few years; however, the incidence of MSVI showed no significant decline [19,20]. Despite the novel developments in the preventive and therapeutic regimens, the cases of early-stage AMD are expected to rise at a brisk pace due to the exponential rise in the aging population and the prevalence of blindness attributable to AMD will increase two- to threefold without treatment. It will be a substantial economic and social burden on health care and will significantly impact the quality of life of the elderly population [21,22].

Aging is briskly becoming an imminent epidemic worldwide, particularly in the industrialized developed nations. As per the predictions of the United Nations, the population of individuals aged over 60 will increase three times, estimated to be 2 billion, and those aged over 80 will increase fivefold by the year 2050 and will constitute 33% of the population in the developed world. The major paradigm shift in the age of the population is a reason for concern for the emergence of health conditions directly correlated with aging [23]. Our review of the literature delineates the risk factors, stages of AMD, and diagnostic and therapeutic modalities to help clinicians understand the impact of the make appropriate clinical decisions to mitigate the morbidity related to AMD.

## Review

Pathogenesis and risk factors

The posterior pole of the retina, known as the macula, undergoes irreversible changes in both dry AMD and wet AMD [24]. The root cause of this disease involves inflammation in the affected area, the buildup of a metabolic byproduct called lipofuscin (LF), the deposition of a yellowish pigment called drusen, and the growth of new blood vessels called neo-angiogenesis. The condition can initially present as small drusen deposits or color changes, progressing to more severe stages with the development of choroidal atrophy or abnormal blood vessel growth known as choroidal neovascularization (CNV) [25,26].

AMD is a condition that predominantly damages two regions of the eye: RPE and BrM, which is a layer of collagen between the RPE and the blood vessels in the choroid. Drusen is a yellowish acellular deposit, made up of polymorphous debris that can be seen under a microscope between the RPE and the inner collagen layer of BrM. The buildup of drusen creates a barrier that restricts the flow of important nutrients and waste products between the choriocapillaris and the neural retina. These drusen are composed of several substances, including plasma proteins, apolipoprotein E (APOE), lipids rich in cholesterol, polysaccharides, glycoprotein, and plasma amyloid P. Studies using immunochemical analyses have shown that these substances are responsible for deactivating the complement system and lead to the formation of membrane attack complexes (MACs) [27].

The worsening of the local inflammatory condition leads to the deterioration of the photoreceptors and RPE. Drusen, which are pigmentary deposits, are commonly classified as *hard* or *soft*. Hard drusen are observed in the eyes of aging individuals, while soft drusen are closely associated with the likelihood and progression of AMD [28,29]. LF, which is also called a cell-aging factor, is a byproduct produced when the phagolysosomes are unable to digest the outer segments of photoreceptors effectively. The protein-lipid membrane holding the liposome becomes less tightly bound, allowing LF to move into the cytoplasm and eventually into extracellular spaces, where it contributes to the formation of drusen. The harmful effects of LF and A2-E, which is a toxic vitamin A dimer, can cause damage to photoreceptors and choriocapillaris, leading to the development of GA [30].

The primary risk factors for AMD include advancing age, smoking, hypertension, atherosclerosis, and oxidative damage caused by exposure to light [31,32]. In addition, genetic factors such as mutations in genes related to the complement system (complement factor H, or CFH, and age-related maculopathy susceptibility 2, or ARMS2) [33,34] and abnormalities in lipid, angiogenic, and extracellular matrix pathways [35] are significant risk factors. Other factors linked to the development of AMD include previous cataract surgery, high body mass index, and elevated levels of plasma fibrinogen [36].

Genetics has a huge influence on the development of AMD, where 103 AMD-related genes and loci have been identified. CHF and high-temperature requirement A serine peptidase 1 (HTRA1) are two major loci associated with AMD. CHF is one of the vital regulators of the complement pathway, and its function is to inhibit the activation of C3 to C3b and the degradation of C3b. Nonfunctional CHF 402H leads to an overactivated complement system, elevating the risk of AMD [37-39]. Polymorphisms in CHF and ARMS2 alleles together account for a 45% risk of developing AMD [40]. Some AMD genes have been linked to lipid metabolism. APOE epsilon 4 carriers have a decreased risk of AMD as it enhances the mobility of cholesterol, lipid, and RPE degradation products across BrM, whereas APOE epsilon 2 increases the risk of AMD by amplifying the expression of vascular growth factor and fibroblast growth factor (FGF) in RPE cells [41-43].

Smoking cigarettes has been linked to increased levels of oxidative stress, lipid peroxidation, and fibrinogen and platelet aggregation all of which have detrimental effects on the body. In addition, cigarette smoking can cause atherosclerosis, which can damage the choroidal arteries and reduce choroidal blood flow. Nicotine, a chemical found in cigarettes, can stimulate the production of VEGF, a proangiogenic factor that promotes angiogenesis and is involved in the development of nAMD. Studies have shown that smoking can also cause inflammation by decreasing complement factor H levels in the blood and activating other inflammatory mediators such as complement C3. Even after 20 years of smoking cessation, former smokers remain at a higher risk of developing AMD, according to research conducted in Rotterdam [44,45].

The retina is among the highest oxygen-consuming tissue of the human body. Oxidized lipoproteins and free radicals are major contributors to tissue stress in the aging retina and act as specific triggers of retinal para-inflammation. This para-inflammatory response can cause microglial activation, migration into the subretinal space, and breakdown of the blood-retinal barrier. Inflammatory cells have been found on the external surface of BrM in the eyes of AMD patients, and proteins associated with immune-mediated processes and inflammation have been identified in the drusen [32]. Additionally, excessive sunlight exposure leads to an increased burden of antioxidant formations [46].

The most frequent cause of blindness among the elderly is wet AMD, which is characterized by the development of CNV beneath the retina. Angiogenesis is activated by several substances, including VEGF; nitric oxide (NO); integrins a5b1, avb3, and avb5; and transforming growth factor (TGFb1) and its receptors, as well as growth factors such as FGFs, including aFGF and bFGF [47].

Multiple studies suggest that individuals with high blood pressure are at an increased risk of developing AMD. This link may be due to the effect of systolic blood pressure on choroidal blood flow. Additionally, the role of hyperlipidemia in AMD development is also noteworthy. Some researchers suggest that high levels of low-density lipoprotein (LDL) and fat consumption may contribute to nAMD, while hypercholesterolemia may be indicative of the onset of GA [24].

While studies on the gender predisposition to AMD have yielded inconclusive results, the incidence of AMD is comparatively higher in females [19,28].

AMD and Alzheimer's disease (AD) have similarities in the formation of abnormal extracellular deposits such as plaques and drusen that are associated with neuronal degeneration. In AD, hallmark features are the accumulation of hyperphosphorylated tau (p-tau) and amyloid-beta (A-beta) proteins in the extracellular space, leading to neuroinflammation, brain iron dyshomeostasis, and delayed neuronal death. These overlapping pathological mechanisms between AMD and AD contribute to their similarity [48].

Types and grading of AMD

There exist multiple grading systems to classify AMD for both diagnostic and prognostic purposes. However, the two main types of AMD that are commonly delineated include dry AMD and wet AMD. Both can then further be divided into subclasses and stages depending on their ocular manifestations and progression [49].

Dry AMD, also known as a non-exudative type of macular degeneration, is characterized by the presence of drusen, which are tiny yellow opaque deposits between RPE and BrM [49]. These extracellular deposits varying in size and density appear as the earliest features of macular degeneration [50]. AMD is mainly categorized into early, intermediate, and advanced forms. The early form of AMD is usually asymptomatic and is only diagnosed clinically on an ocular examination. The initial stage (stage 1) of AMD is normal aging alterations with drusen size <63 µm, also referred to as drupelets, and no pigment abnormalities. The size and extent of drusen in early AMD (second stage) is between 63 and 124 μm diameter and has no associated RPE cell irregularity. Large drusen of size ≥125 μm diameter are seen in intermediate AMD (third stage) with associated RPE abnormalities [51,52]. The presence of drusen is often accompanied by alterations in RPE, resulting in focal areas of hypopigmentation or hyperpigmentation. These RPE pigmentary changes are predictors for the risk of progression to advanced stages and, therefore, contribute to a potential visual loss [53]. A much common grading mentioned and used by many researchers and clinicians defines the advanced form of dry AMD as the occurrence of GA, which involves atrophy of choriocapillaris associated with progressive irreversible degeneration of photoreceptor cells [54].

The other spectrum of AMD encompasses the neovascular form or exudative/wet type of AMD, where new blood vessels proliferate into the sub-RPE or subretinal spaces [50]. The development of CNV causes leakage of abnormal vessels resulting in hemorrhage and even serious complications such as RPE detachment, leading to rapid sight loss [55]. Although rare, serous pigment epithelial detachment (PED) can progress to life-threatening visual loss due to the accumulation of fluid in subretinal spaces causing tearing of RPE and secondary atrophy of the outer retina [56].

The process of degeneration and cell death in AMD is really slow and usually, patients experience no symptoms in the early stages. Clinical manifestations are typically assessed through macular morphology, and no genetic biomarkers are routinely used to screen for the disease [57]. In addition to morphological manifestations of macular changes in AMD, the visual assessment requires the establishment of valid functional endpoints. The measurement of best-corrected visual acuity (BCVA), the ability to distinguish details of an object at a given distance after correction of refractive errors, is often utilized as a valid functional parameter to monitor deterioration in sight and, hence, quality of life [58]. The usual outcome of the non-neovascular type is slow-but-steady progressive vision deterioration, which spans months to years. The first clinical sign might be drusen only, but certain peculiar structural characteristics of drusen are associated with rapid progression to advanced stages of AMD [59]. The definitive outcome of the non-exudative form is an irreversible distortion of RPE and GA [60]. But, as soon as it is complicated by exudative changes, an abrupt loss of visual potential occurs, which substantially threatens the quality of life, even over days to weeks [56]. Irrespective of a variant of the disease, whenever the fovea is involved in either atrophic or exudative changes, severe visual impairment can occur due to central vision loss [55].

Diagnosis

**Figure 3:**
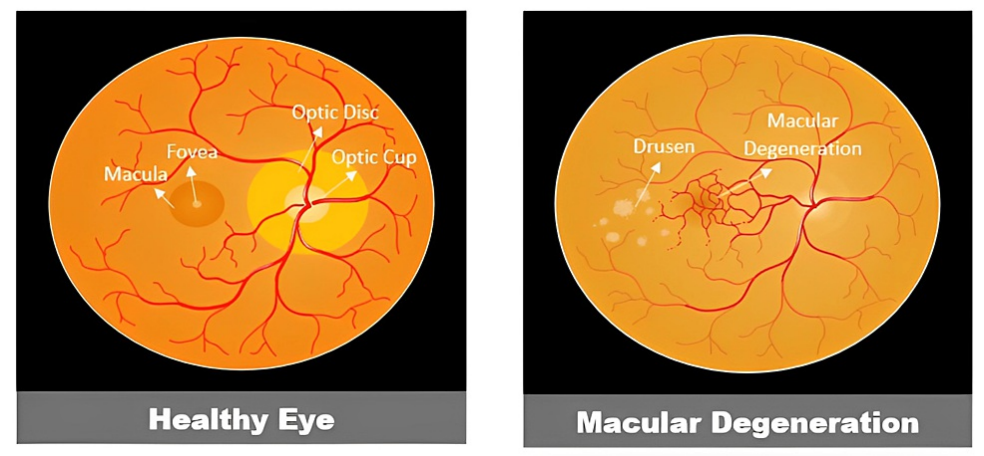
Fundoscopic examination healthy eye versus macular degeneration. Parts of the figure were drawn using pictures from Servier Medical Art. Servier Medical Art by Servier is licensed under a Creative Commons Attribution 3.0 Unported License (https://creativecommons.org/licenses/by/3.0/).

A dilated fundus examination should be performed on anyone aged over 55 years to check for macular degeneration (Figure 3) [61]. To diagnose AMD, the examiner should look for drusen deposits, pigmentary alterations, GA, hemorrhage, fluid exudation, scarring, and fibrosis. The distribution, size, and quantity of drusen are all taken into consideration. To rule out concurrent ocular pathologic disorders, a thorough eye examination is performed. Although the examination may account for the majority of the disease staging, the use of a range of imaging methods is now seen to be crucial for correlating examination findings and directing therapy [62]. The rate of technological development in ophthalmology is astounding, and during the past two decades, there has been substantial evolution in retinal imaging modalities.

In the usual clinical setting, the Amsler grid and preferential hyperacuity perimetry (PHP) are two predominantly used tests for the diagnosis of AMD [63]. The Amsler grid is a square-shaped grid that is used to identify or track scotomas or metamorphopsia (visual distortion) affecting the central visual field in a variety of macula and optic nerve head disorders. It serves as a low-cost home-monitoring self-assessment tool for the early diagnosis of metamorphopsia brought by wet AMD. A meta-analysis conducted on the Amsler grid, however, showed low sensitivity of 67% (95% confidence interval [CI] 51%-79%), and hence, patients are encouraged to undergo ophthalmic examinations regularly regardless of the Amsler grid results [64]. PHP measures the severity of visual abnormalities, including metamorphopsia and scotoma within the central 14° of the visual field by utilizing the very sensitive visual function of hyperacuity. PHP has shown high sensitivity (82%) and specificity (88%) in differentiating recent onset CNV and intermediate forms of AMD. Additionally, PHP has been proposed as a technique for monitoring the effectiveness of photodynamic therapy and anti-VEGF therapy in treating nAMD [65,66].

Historically, the gold standard for determining CNV in AMD has been fluorescein angiography (FA). Fluorescein dye is injected into a patient's vein, and over several minutes, pictures of the chorioretinal circulation are recorded. This invasive imaging modality may be used to identify the existence of leakage from various kinds of neovascular lesions. An analogous technique called indocyanine green angiography (ICG) could be used in specific circumstances. Indocyanine green dye is injected during ICG procedures, which assists in assessing the choroidal circulation and enables the detection of occult CNV lesions [66].

The knowledge and treatment of AMD have been transformed by optical coherence tomography (OCT), a popular noninvasive technology that allows for an in-depth display of each of the retinal layers [66]. By providing a thorough cross-sectional picture of the 10 retinal layers and underlying choroid using light rather than sound waves, OCT is similar to ultrasound in that it enables the identification of the particular layers affected by AMD. The photos allow the doctor to more accurately describe the disease stage and CNV activity while also assisting in the distinction between wet AMD and dry AMD. OCT may be compared longitudinally to evaluate therapy response and further management. OCT may reveal fluid inside and under the retina that is present in wet AMD [67-69].

OCT angiography (OCT-A) is a relatively contemporary imaging modality. OCT-A is a noninvasive innovative technique that enhances the ability to see the intricate vascular network of the choroid. This method assists in the comprehension of the microvascular alterations that take place in nAMD when CNV lesions are present. Moreover, OCT-A can be used to detect neovascularization early, enabling more thorough surveillance and, if necessary, earlier intervention. OCT-A has replaced FA and ICG in most clinical scenarios [70].

However, OCT is not sensitive enough to detect early signs or identify patients at higher risk of disease progression due to inferior spatial resolution and inadequate intrinsic details from the retinal and RPE cells and microvasculature. Therefore, new novel diagnostic tools are emerging to achieve higher reliability in identifying specific signs of disease progression. Adaptive optics (AO) retinal imaging is currently under study as it provides higher resolution and delineates intricate patterns of microstructural aspects of photoreceptor cells, microvasculature, and retinal nerve fibers. Resonance Raman spectroscopy (RRS) is another promising modality that precisely measures the levels of carotenoid and xanthophyll concentration in the macular region of the human retina [71-74].

Prevention

Lutein and its isomer zeaxanthin are the major constituents of the macular pigment in the human retina, with the highest concentration found in the foveal region. The main function of these carotenoids is to protect the photoreceptor’s membrane system from photo-induced and radiation damage [75,76].

The Age-Related Eye Disease Study (AREDS) indicated a 25% decrease in the development of progressive AMD and defined criteria for vitamin supplementation. Zinc, vitamin C, vitamin E, lutein, and zeaxanthin are the main components of the current supplements. Because of the increased risk of lung cancer in smokers, beta-carotene was substituted by lutein and zeaxanthin in the AREDS2 formulation. Supplementation is advised for individuals with intermediate AMD or early AMD in one eye and advanced AMD in the other eye. However, it has not been demonstrated to be helpful for those with early AMD in both eyes. Supplementation is also not advised for the prevention of AMD in the general population or for those who have a positive family history of the disease [77]. The eye disease case-control study also depicted a decline of 40% in the risk of advanced AMD among individuals who consumed >6 mg of carotenoid per day [78]. Currently, research is being conducted to develop a novel scleral iontophoresis device to deliver liquid carotenoid formulations directly into the retina [71,74]. 

All patients should make lifestyle changes, including those who have early AMD in one or both eyes. Together with weight reduction and smoking cessation, these alterations may involve dietary adjustments to include foods high in antioxidants and the omega-3 and omega-6 fatty acids present in fish. Lipid profile and blood pressure are two modifiable risk factors. Although there is mixed data linking UV light exposure to AMD development, reducing sun exposure is another possible lifestyle modification [79-81].

Low-vision aids

Patients with advanced AMD who have considerable vision loss must have access to low-vision aids to maximize their degree of daily-life visual function. Magnification lenses were the main choice up until recently. Although they are a useful tool, persons with impaired eyesight also have access to more recent modalities. A recently developed optical identification tool called the OrCam, is put on a frame and used to help identify faces, text, and other items [82]. In addition, modern smart gadgets contain many functions that help people with impaired vision, such as voice assistance, brightness adjustment, bigger text, and dictation, to mention a few. Investigations on implantable micro-telescope technologies involve having suitable individuals go through a process that includes both cataract removal and telescope implantation [5]. For the right patient, these implants might be a promising choice as they act as a visual simulator. Patients with vision loss brought on by AMD or another eye condition may benefit greatly from being sent to a low-vision specialist, which can also have a beneficial impact on other non-ocular factors, including fall risk and mental health.

Treatment of AMD

Anti-VEGF

Before the advent of powerful anti-VEGF medications for ophthalmic treatment, a diagnosis of wet AMD indicated inevitable, irreversible central vision loss. Success in the initial wave of nAMD therapies, which included laser photocoagulation, photodynamic therapy, irradiation, and angiostatic steroids, was often characterized as the avoidance of progression to moderate-to-severe visual acuity loss due to nAMD, but not an adequate improvement in visual function [83,84]. Anti-VEGF drugs work by reacting directly with VEGF or with VEGF receptors. This family of medications has surpassed all past therapy approaches, allowing the best corrected visual acuity (BCVA) elevation from baseline and visual function maintenance and is currently the preferred treatment for wet AMD [85]. Dry AMD, though significantly more frequent, accounts for just one-fourth of AMD-related instances of severe vision loss and no preventive or treatment alternatives are available for it [86].

Ranibizumab, bevacizumab, aflibercept, and brolucizumab are currently available intravitreal VEGF inhibitors. Dosing regimens for ranibizumab and bevacizumab are every four to eight weeks, every 12 weeks for aflibercept, and every eight to 12 weeks for brolucizumab. Pro re nata (PRN) or treat and extend are two alternative anti-VEGF dosage methods. These procedures begin with an induction period of a set monthly dose, generally lasting three months. PRN dosage entails monthly assessments during which injections are either administered or delayed based on OCT findings of active exudation or stable illness. Even if no disease activity is noted, the treat-and-extend method entails intravitreal injection at each appointment. If the retinal fluid is shown to be suppressed or cleared on OCT, the time between visits is increased by a predetermined period. If fluid recrudescence is discovered, an injection is given and the time between visits is decreased [87].

Designed Ankyrin Repeat Proteins (DARPins)

Natural ankyrin repeat proteins, which are among the most prevalent binding proteins encoded in the human genome, are the source of DARPin molecules [88]. The therapeutic effectiveness of abicipar-polyethylene glycol (Pegol), a 34-kDa recombinant PEGylated DARPin that potently inhibits VEGF-A, is now being studied as a treatment for diabetic macular edema and nAMD [89,90].

Abicipar has a lower molecular weight, a 90-fold greater affinity for human VEGF-A165, and roughly a twofold longer intraocular half-life than ranibizumab [91]. Abicipar (1 or 2 mg) had a sustained benefit equivalent to ranibizumab (0.5 mg) in phase 2 Registration, Evaluation, Authorization, and Restriction of Chemicals (REACH) trial in terms of mean BCVA improvement and central retinal thickness, with no significant safety issues [92]. With six to eight injections of abicipar, compared to 13 injections of ranibizumab at week 52 of the phase 3 Sequoia and Cedar studies, both abicipar regimens (every eight weeks and every 12 weeks) proved to be superior to monthly ranibizumab for achieving stable vision [91]. The visual advantages from year one were sustained in year two similarly in all abicipar dose frequencies [93]. Researchers assessed the safety and therapeutic efficacy of abicipar using a modified manufacturing technique in phase 2b Maple trial to reduce inflammation-inducing contaminants in the formulation [93,94]. The findings revealed no endophthalmitis or retinal vasculitis complications and a 1.6% prevalence of severe intraocular infections [93].

Recombinant Adeno-Associated Virus (rAAV) Gene Therapy

Long-term control of AMD is made possible by the injection of AAV vectors and the subsequent expression of VEGF-inhibitory molecules by recipient cells. A phase 1 trial for nAMD found that a single subretinal injection of an rAAV particle containing the Fms-Related Receptor Tyrosine Kinase 1 (FLT1) transcript, which encodes a naturally occurring VEGF inhibitor, was safe [95], with rAAV.sFLT-1 (soluble FLT-1) was primarily contained within the target tissue and resulted in no significant ocular or systemic adverse effects [96]. A 52-week phase 2 clinical study comprised 32 patients who were randomly assigned to receive ranibizumab PRN with or without subretinal rAAV.sFLT-1 gene therapy revealed no significant differences between the groups in BCVA or center point thickness [96]. The authors discovered minimal effectiveness in terms of BCVA increases and fluid decrease in the phase 1/2b study's three-year follow-up. They noted that the effects of rAAV.sFLT-1 may have been masked because the sample size was small and the study was originally planned to examine safety and included non-treatment-naive individuals [51].

Thermal Laser Photocoagulation

Researchers discovered that continuous-wave thermal laser photocoagulation could reduce drusen deposits in the outer retinal layers decades before anti-VEGF therapies became available for nAMD. However, subsequent clinical trial results showed that laser-induced drusen elimination did not prevent progression to advanced AMD [97]. Moreover, laser photocoagulation was frequently related to worsening BCVA, CNV recurrence, and scarring [98]. Researchers have recently devised a short-pulse laser treatment that offers enough energy to ablate drusen but not enough to cause a thermal burn and subsequent retinal necrosis. The therapeutic and placebo groups in a three-year randomized study of subthreshold nanosecond laser therapy showed a similar time to late AMD onset [97].

Clustered Regularly Interspaced Short Palindromic Repeats (CRISPR)-Associated Protein 9 (Cas9)

The CRISPR-Cas9 enzyme cuts DNA strands like a pair of molecular scissors [99]. Researchers are now assessing the viability of a CRISPR-Cas9-based genome editing strategy given with an AAV vector to permanently reduce VEGF in the RPE layer of eyes with nAMD, thereby minimizing angiogenesis. The idea of avoiding the need for periodic anti-VEGF injections is appealing. This method reduced VEGF-A by 26% and suppressed CNV by 31% in a mouse model of laser-induced CNV. In contrast to intravitreal therapeutic approaches, CRISPR may be genetically programmed to target specific cell types while minimizing systemic side effects. Although the CRISPR technique shows promise as a treatment for nAMD, it must be evaluated with great caution since CRISPR-based genome editing is susceptible to off-target consequences and is irreversible [100].

Brimonidine

In cultured cells and animal models, brimonidine is an alpha-2 adrenergic agonist with cytoprotective and neuroprotective effects. Researchers examined the Brimonidine Drug Delivery System (Brimo DDS), a biodegradable intravitreal implant, for its ability to stop the development of GA in a 24-month phase 2 experiment. In contrast to placebo, patients were given 2 dosages of brimonidine (132 or 264 µg). At 12 months, the higher dosage implanted group's GA growth had decreased by 28% in comparison to the placebo group. Brimo DDS produced extra advantages in individuals with GA lesions 6 mm^2^ or greater at entry into trial and decreased the GA progression rate at 12 months [101]. Patients in the phase 2b BEACON trial received 400 µg of brimonidine intravitreally every three months from baseline to month 21, and the impact on the GA lesion size was evaluated at month 24 [102]. At months 24 and 30, the findings demonstrated 7% and 11% reductions, respectively, in the size of GA lesions [103].

CK41061

A new dry AMD therapeutic candidate, CK41016, which can prevent RPE cell death, was disclosed by Choi et al. [104]. In both rat and rabbit models, the scientists used intravenous and topical (eye drop) routes to administer CK41016, which functions as an antioxidant and increases the blood flow in retinal tissue. They discovered that the tissue distribution and pharmacokinetic patterns varied by species. Before clinical trials can be started, the characteristics of this chemical must be further assessed given the species discrepancy. For dry AMD, topically applied medications must reach the rear of the eye, although this is challenging with eye drop formulations [105].

Regenerative Therapies

Although gene therapies and optogenetic techniques are being researched, high-resolution vision restoration is unlikely to be made possible by these methods shortly. Cellular regeneration treatment is an additional choice. With severe AMD, the inner retina is still functional, thus the idea of removing the damaged outer retina and restoring it with healthy tissue is appealing. RPE transplantation has been shown in a few clinical situations when photoreceptor degeneration had not yet started to theoretically restore the function of this cell layer and save failing photoreceptors. Pluripotent stem-cell-derived cells are thought to be the most promising of the several cell sources that have been evaluated as options. Recent primordial and early-phase clinical studies using these cells to replace RPE have produced acceptable safety profiles and possible effectiveness. Moreover, evidence from preclinical research suggests that photoreceptor replacement might restore visual function even in an outer retina that has completely deteriorated [106].

## Conclusions

Our review of the literature highlights that the aging population will constitute a huge proportion of the community in the coming decades and age-related disorders are an imminent matter of concern. The quality of life of the elderly population is significantly impacted by age-related macular degeneration, which is a massive worldwide health concern. The ability to read, drive, identify people, and carry out basic daily duties are all impacted by central vision loss due to AMD. The phenotypic manifestation of the illness varies significantly, ranging from early or intermediate AMD, which may have few symptoms, to late disease, which may cause the patient to experience visual distortion, diminished central visual acuity, scotomas, and complete loss of central vision. Specialists in retinal imaging are now able to more precisely assess the severity of the disease and make an earlier diagnosis of AMD owing to multimodal imaging of the retina's ultrastructure. Detailed funduscopic examination among asymptomatic patients to identify drusen is slowly gaining acceptance as a screening tool, while optical coherence tomography is a vastly popular diagnostic study among medical practitioners.

The use of certain vitamins and carotenoids, specifically lutein, in supplements has shown encouraging results in halting the progression of advanced AMD; however, it did not completely mitigate the pathology. Certain lifestyle changes, including smoking cessation, weight loss, and cholesterol reduction, can be promoted as potential risk factor modification. Advanced neovascular AMD patients' visual outcomes have enhanced as a result of the development of anti-VEGF medication. However, anti-VEGF medication does not always work on every patient; it also tends to lose its effectiveness with time, and the requirement of frequent injections places an enormous burden on the healthcare system, patients, and caregivers. Research is being conducted to decrease this burden and enhance the benefit of VEGF suppression and provide new therapeutic techniques, such as gene and regenerative therapy. Unfortunately, there are limited therapeutic options for patients with GA due to advanced AMD.

The authors advocate that policies and guidelines for people aged above 50 years for screening AMD in the earlier stage should be materialized to curtail social and financial burdens in the future. It is imperative to share the knowledge of modifiable risk factors and beneficial supplements at the public level to help acknowledge the disease and mitigate AMD-related morbidity. There is a dire need to conduct research at a brisk pace to develop modalities to diagnose AMD precisely at an earlier stage and treat or potentially reverse the progression of the disease to enhance the quality of life.
